# Comparison of the efficacy of expandable interbody fusion cage (EXP-IFC) and non-expandable interbody fusion cage (NE-IFC) in MIS-TLIF for lumbar degenerative diseases: A systematic retrospective study on 62 patients

**DOI:** 10.3389/fsurg.2022.1008171

**Published:** 2022-10-18

**Authors:** Chen Chen, Qiang Li, Wei Wang, Chunlei Ji, Yi Kang, Chaoyu Wang, Hongyi Zhang, Ming Zhang, Hengxing Zhou, Haoyu Feng, Shiqing Feng

**Affiliations:** ^1^ Shanxi Bethune Hospital, Shanxi Academy of Medical Sciences, Tongji Shanxi Hospital, Third Hospital of Shanxi Medical University, Taiyuan, China; ^2^ Tongji Hospital, Tongji Medical College, Huazhong University of Science and Technology, Wuhan, China; ^3^ Department of Orthopaedics, Qilu Hospital, Shandong University Centre for Orthopaedics, Advanced Medical Research Institute, Cheeloo College of Medicine, Shandong University, Jinan, China; ^4^ Department of Orthopaedics, International Science and Technology Cooperation Base of Spinal Cord Injury, Tianjin Key Laboratory of Spine and Spinal Cord, Tianjin Medical University General Hospital, Tianjin, China

**Keywords:** expandable interbody fusion cage, lumbar fusion, MIS-TLIF, radiographic parameters, lumbar degenerative disease

## Abstract

**Objectives:**

To investigate the clinical and radiographic outcomes of EXP-IFC in single-level MIS-TLIF.

**Methods:**

This study included patients aged ≥18 years who received a single-level MIS-TLIF procedure with at least 1 year of follow-up. Outcome measures: clinical features, preoperative and neurological complications. Imaging analysis included disc height (DH) restoration, surgical and contralateral side foraminal height (FH), lumbar lordosis angle (LL), segmental lordosis (SL). Visual analog scale (VAS) score for low back pain (VAS-LBP) and leg pain (VAS-LP), Oswestry Disability Index (ODI) and Japanese Orthopaedic Association (JOA) score were used to evaluate clinical outcomes. Statistical analysis was performed using independent sample t-test and sample t-test. The significance was set to *p* < 0.05 in univariate analysis.

**Results:**

A total of 62 patients undergoing single level MIS-TLIFs between January 2017 and January 2019 were included, with 32 NE-IFC 46.9% female, mean age 54.86 ± 11.65, mean body mass index (BMI) 24.59 ± 3.63) and 30 EXP (40% female, mean age 58.32 ± 12.99, mean BMI 24.45 ± 2.76) with no significant differences in demographics. There were no significant differences between two groups in Operative time (OT), Estimated blood loss (EBL) and Length of stay (LOS). No significant differences were found in VAS-LBP, VAS-LP, JOA and ODI in post-operation and the last follow-up between the two groups. The imaging outcome demonstrated that the mean increase in DH was significantly greater for the patients with EXP-IFC than those with NE-IFC group at 1 year follow-up (8.92 ± 0.51 mm EXP-IFC vs. 7.96 ± 0.96 mm NE-IFC, *p* < 0.001). The mean change in FH of operative and contralateral sides were observed to be significantly higher for the patients with EXP-IFC at 1 year follow-up (operative side:17.67 ± 2.29 mm EXP-IFC vs. 16.01 ± 2.73 mm NE-IFC, *p* = 0.042; contralateral side:17.32 ± 2.26 mm EXP-IFC vs. 16.10 ± 2.32 mm NE-IFC, *p* < 0.001), but changes in LL and SL were not significantly different. At the last follow-up, we did not find any significant difference in the fusion rate between the two groups.

**Conclusion:**

Our results indicated that there may be no significant difference in short-term clinical outcomes between EXP-IFC and NE-IFC, but the use of EXP-IFC in MIS-TLIF can provide a significant restoration of disc height, and neural foraminal height compared to NE-IFC.

## Introduction

Lumbar degenerative diseases are common in the elderly and often cause pain, disability, and poor quality of life (1). With the prevalence of lumbar degenerative disease rising in keeping with the aging population, surgical treatment of lumbar degenerative conditions has shown a dramatic increase. Minimally invasive transforaminal lumbar interbody fusion (MIS-TLIF) is a minimally invasive technique and widely used in the lumbar degenerative diseases (2, 3). In MIS-TLIF, direct unilateral laminectomy and resection of the inferior articular process into the intervertebral space are performed to decompress the nerve roots. In the procedure, an autogenous cancellous or allogeneic bone fusion cage were placed into the intervertebral space to provide anterior column support, restore the height of intervertebral space so as to achieve the effect of nerve root canal decompression and provide mechanical stability for the lumbar spine (4, 5).

**Figure 1:**
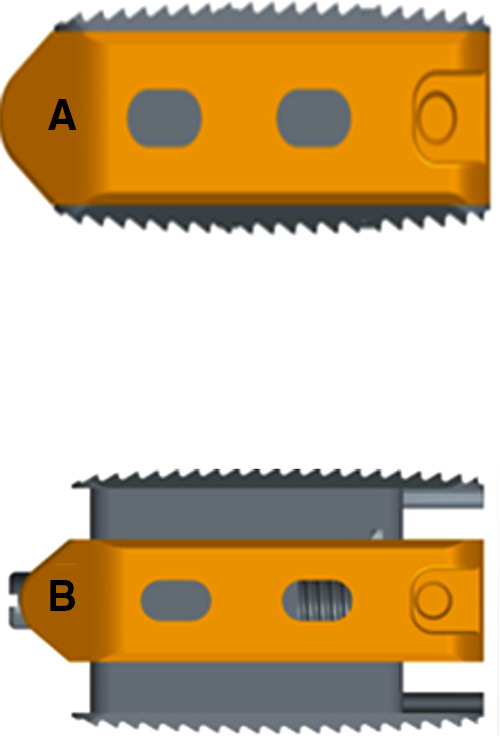
(**A**) Before distraction, the height was 8 mm, which was significantly smaller than the distance between the outlet nerve root and the upper edge of the inferior pedicle. (**B**) After stretching, the height can be stretched to 9, 10, 11, 12, 13 mm, which can be selected according to the specific situation.

In recent years, it is suggested that lumbar spine surgery should be performed on the basis of complete decompression to minimize the damage to the stable structure of the spine (6). With the reduction of incision and access area, the traditional non-expandable interbody fusion cage (NE-IFC) can not meet the needs of present minimally invasive fusion. In the process of implanting, NE-IFC will destroy the lateral surface of endplate and cortical bone because of striking hard, this may adversely affect the stability and fusion rate of the fusion device (7). Expandable interbody fusion devices (EXP-IFC) are a good option, as they collapse into the disc space to reduce damage to the upper and lower endplates, at the same time, it can reduce the amount of blood loss and become a less invasive alternative (8, 9). (Figure 1). In the MIS-TLIF operation, the application of EXP-IFC can not only obtain satisfactory clinical outcomes, but also effectively restore DH, FH, and reduce lumbar spondylolisthesis and improve lumbar lordosis angle (LL) and segmental lordosis angle(SL) (10).

However, there is still controversy concerning the ability of EXP-IFC to restore sagittal alignment and correction of radiographic parameters (11, 12). With increasing evidence highlighting the association of restoration and maintenance of spinopelvic parameters with improved prognosis after spinal surgery, there is interest in applying these concepts to MIS TLIF with EXP-IFC as well (13). The various studies in MIS-TLIF that observed improvement in EXP-IFC reported information based on short-term follow-up time, or only reported clinical outcomes in EXP-IFC implant patients, but did not establish a comparative arm (8, 13, 14).

The purpose of this study was to compare the clinical outcomes and restoration of LL, SL, DH and FH in patients who had underwent MIS-TLIF with either NE-IFC or EXP-IFC one year after surgery.

## Materials and methods

### Patients

A single-center retrospective analysis of adult patients (≥ 18 years old) undergoing selective single-level MIS-TLIF from January 2017 to January 2019 was conducted. Patients met the following inclusion criteria: (1) a minimum 1 year follow-up (2) diagnosis of single-level lumbar degenerative disease diagnosed by MRI; (3) no previous fusion history; (4) surgery performed by the same group of doctors; (5) the use of expandable and non-expandable fusion cages. Exclusion criteria were as follws: (1) severe degeneration in adjacent segments; (2) lumbar spondylolisthesis and severe bony spinal canal stenosis; (3) multilevel disease; (4) history of lumbar fracture, infection, deformity, tumor and severe osteoporosis; (5) incomplete clinical and imaging data.

This study was approved by Medical Research Ethics Committee of Shanxi Bethune Hospital and informed consent was waived due to the retrospective nature of the study.

### Surgical technique

In this study, the choice of fusion cage type was based on the optimal height according to preoperative imaging data. The height of fusion cage should be slightly larger than the preoperative intervertebral height by 20%, the length of the cage should be approximately 4 mm shorter than the anteroposterior diameter of the vertebral body. The cage is made of titanium alloy. Its height is between 8 cm and 13 cm and can be adjusted to the right position according to the surgical needs (Figure 1). All operations were performed by the same chief surgeon who has more than 30 years of experience. Under general anesthesia, the patient was placed in prone position and the abdomen was suspended. The G-arm was located in the corresponding intervertebral space of the operation. The average length of the 4.3 cm incision was made at the outer edge of the pedicle on one side. Subsequently skin, subcutaneous and deep fascia were cut layer by layer, and the channel guide core was inserted. The G arm was located in the corresponding intervertebral space and the dorsal side of the facet process, then expanded to the working cannula step by step, expanded the channel, and installed the light source. The bone of the intervertebral foramen on one side of the corresponding intervertebral space was removed to protect the corresponding nerve root. The nerve root and dural sac were opened by the subaxillary nerve retractor, and one side of the lateral process of intervertebral disc in the corresponding vertebral space was protruded. After careful separation of the dural sac, the corresponding intervertebral disc was removed, the upper and lower endplates and hyperplastic osteophytes were treated, and a lumbar cage was implanted in the intervertebral space, which was filled with autogenous bone fragments. At this time, the nerve root is relaxed and the range of activity is increased. Under the guidance of G-arm, the pedicle screw guide needle was percutaneously inserted into the corresponding vertebral space, and the display position was good; four hollow pedicle screws were screwed in turn along the guide needle, and the G-arm showed a good position again; a fixed rod with physiological radians was installed and confirmed by G-arm fluoroscopy, adjust and tighten each screw, indwelling negative pressure drainage tube. Antibiotics were routinely used for 1 day post-operation to prevent infection. The drainage tube was removed 1–2 days post-operation. Methylprednisolone was given to protect the nerve and mannitol was given to relieve nerve root edema for 3 days. The straight leg was raised actively or passively on the first day post-operation to prevent nerve root adhesion. Regular review.

### Data collection

Data on demographic, clinical characteristics and outcomes were obtained from the patients' electronic medical records. Demographic data included age, body mass index (BMI) and gender. Surgical parameters included operation time, estimated blood loss (EBL) and length of stay (LOS).

The visual analogue scale (VAS) for low back pain (LBP) and leg pain (LP), the Oswestry disability index (ODI) and the modified Mac Nab standard were used to evaluate the clinical effect. Japanese Orthopaedic Association (JOA) score was used to evaluate the improvement of neurological function (15). An improvement rate of 100% for JOA is considered as all healed, 60%–100% as remarkable; 25%–60% as effective and <25% as ineffective. ODI was used to assess the improvement of clinical function in patients (16), with a total score ranging from 0 (no disability) to 50 (full disability). The intensity of waist and leg pain were measured using VAS scores on a 10-cm horizontal line, with 0 for no pain, 0 to 2 for comfort, 3 to 4 for mild pain, 5 to 6 for moderate pain, 7 to 8 for severe pain, 9 to 10 for extreme pain and 10 for severe pain (17).

The radiographic parameters included disc height (DH), foraminal height (FH), segmental lordosis (SL) and lumbar lordosis angle (LL) for each surgically treated level. DH was defined as the average of height of anterior edge of upper and lower vertebral body and height of posterior edge of vertebral body in responsible space; FH was defined as the vertical distance between the lowest edge of pedicle of upper vertebral arch and the highest edge of lower vertebral arch of responsible level; SL was defined as the angle between the superior and inferior endplates composing the disk space; LL was defined as the angle between the lower endplate of the L1 body and the sacral plate. Standard lateral lumbar x-ray examinations were performed pre-operatively, post-operatively and at 1 year follow-up. All these parameters were measured by Surgimap Spine Nemaris Inc (Version: 2.2.13.1). Two experienced orthopaedic surgeons who were not involved in the study measured the data on lumbar x-ray images and recorded the average of the measurement results.The fusion was determined by lumbar imaging (there were continuous trabeculae between the implant and the host bone for obtaining bone fusion, and the failure of fusion was characterized by pseudarthrosis and light band around the implant) (18).

### Statistical method

IBM SPSS Statistics Grad Pack 27.0 (IBM SPSS, Chicago, IL, USA) was used for analysis. Measurements following a normal distribution are expressed as the mean ± standard value $\bar{x}\pm s$. Comparisons of age, operative duration, intraoperative bleeding volume, postoperative drainage, JOA score, VAS score, ODI and radiographic parameters between the expandable and non-expandable patients were performed by group-design t-test; independent t-tests were used to compare the above pre- and post-operative indices. The statistical significance of univariate analysis in this study was set at *p* < 0.05.

## Results

### Cohort overview

This study included 62 patients undergoing single-level MIS-TLIF operations consisting of 32 NE-IFCs and 30 EXP-IFCs. There was no difference in latest follow-up time between EXP-IFC and NE-IFC group, respectively (14.35 ± 1.98 months vs. 13.63 ± 1.67 months; *p* = 0.230). For the patients with NE-IFC, 54.6% were female, the mean age was 54.86 ± 11.65 years old, the average BMI was 24.59 ± 3.63. In the EXP-IFC group, 48% were female, the mean age was 58.32 ± 12.99 years old, with an average BMI of 24.45 ± 2.67. All cases were followed up for 1 year after operation to meet the standard of osseous fusion. At the 1 year follow-up, the bone fusion rate was 93.8% in NE-IFC group and 96.7% in EXP-IFC used group with 1 case not achieving osseous fusion, according to x-ray.

### Comparison of baseline and surgical parameters in patients with expandable and non-expandable cages

There were no significant differences in demographics between the two groups, as shown in Table 1. The majority of cages were implanted at L4/L5 (53.33% EXP-IFC vs. 56.25% NE-IFC; *p* = 0.725) followed by cages implanted at L5/S1 (40% EXP-IFC vs. 37.5% NE-IFC; *p* = 0.687) and those at L3/L4 (6.67% EXP-IFC vs. 6.25% NE-IFC; *p* = 0.824).

**Table 1:** Comparison of demographic and clinical characteristics between patients instrumented with EXP-IFC and NE-IFC

Characteristics	Non-expandable	Expandable	*p* value
*N* = 62	32	30	
Sex, no.			
Male	17	18	
Female	15	12	
Mean age ± SD (yrs)	54.86 ± 11.65	58.32 ± 12.99	0.38
BMI (kg/m^2^)	24.59 ± 3.63	24.45 ± 2.76	0.939
Follow-up time (months)	14.35 ± 1.98	13.63 ± 1.67	0.23
Fusion	30/32	29/30	
Level of fusion			
L3-4	2 (6.25%)	2 (6.67%)	0.824
L4-5	18 (56.25%)	16 (53.33%)	0.725
L5-S1	12 (37.5%)	12 (40.0%)	0.687

Bold values are statistically significant at *p* < 0.05.

When the entire cohort was analyzed, the use of EXP-IFC had no significant effect on length of stay (11.57 ± 5.06 days for EXP-IFC vs. 10.57 ± 3.09 days for NE-IFC; *p* = 0.448) and EBL (147.37 ± 109.57 ml for EXP-IFC vs. 133.33 ± 100.12 ml for NE-IFC; *t* = 0.404, *p* = 0.689). No significant difference in operative time between the two groups was also observed (Table 2).

**Table 2:** Comparison of the general situation of operation between patients instrumented with EXP-IFC and NE-IFC.

Group	Non-expandable	Expandable	*p* value
Operative time (h)	2.36 ± 0.67	2.86 ± 1.10	0.103
Estimated blood loss (ml)	133.33 ± 100.12	147.37 ± 109.57	0.689
Length of stay (days)	10.57 ± 3.09	11.57 ± 5.06	0.448

Bold values are statistically significant *p* < 0.05.

### Evaluation of clinical efficacy

NE-IFC and EXP-IFC showed no significant differences in VAS-LBP, VAS-LP, JOA and ODI in pre-operation and post-operation (*p* > 0.05). In all patients, the post-operative clinical function scores improved compared with the pre-operative scores, and these improvements lasted until the last follow-up. During the follow-up period, none of the patients required reoperation. No other differences in clinical function scores were observed between the EXP-IFC and NE-IFC groups (Table 3).

**Table 3:** Comparison of the clinical efficacy at pre-operation, post-operation and last follow-up between patients instrumented with EXP-IFC and NE-IFC.

Outcomes	Non-expandable	Expandable	*p* value
VAS-LBP			
Pre-operation	5.59 ± 1.87	5.37 ± 1.67	0.412
Post-operation	3.15 ± 1.78	3.95 ± 1.58	0.204
Last follow-up	2.45 ± 1.22	2.55 ± 1.12	0.102
VAS-LBP on the operative side			
Pre-operation	6.34 ± 1.79	6.15 ± 1.84	0.512
Post-operation	3.11 ± 1.76	3.21 ± 1.62	0.234
Last follow-up	1.85 ± 0.92	1.82 ± 0.88	0.354
VAS-LBP on the contralateral side			
Pre-operation	2.12 ± 0.97	2.35 ± 0.77	0.324
Post-operation	1.79 ± 0.84	1.77 ± 0.86	0.214
Last follow-up	1.33 ± 0.65	1.23 ± 0.61	0.102
JOA			
Pre-operation	9.51 ± 2.11	9.81 ± 2.81	0.712
Post-operation	27.98 ± 3.35	28.99 ± 3.47	0.301
Last follow-up	20.5 ± 3.89	20.9 ± 3.96	0.124
ODI			
Pre-operation	28.24 ± 2.23	27.22 ± 0.85	0.612
Post-operation	15.21 ± 1.24	16.19 ± 1.12	0.215
Last follow-up	3.65 ± 1.25	3.74 ± 1.18	0.134

* Bold values are statistically significant *p* < 0.05.

### Radiographic comparison of patients with expandable and non-expandable cages

**Figure 2:**
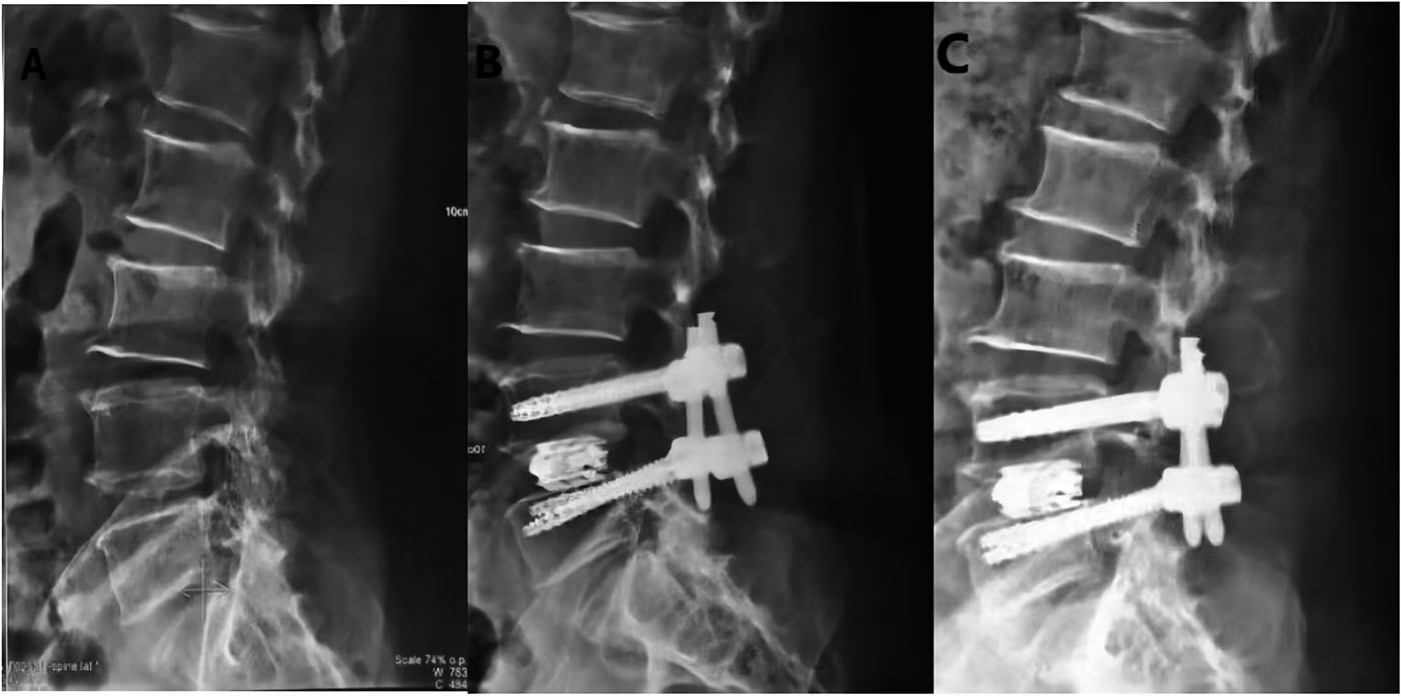
Design of EXP-IFC for anterior and Lateral display of Lumbar vertebrae after MIS-TLIF. (**A**) Preoperative lateral radiograph. (**B**) Postoperative lateral radiograph (1 week); (**C**) Postoperative lateral radiograph (1 year).

Radiographic analysis showed that patients who use EXP-IFC in MIS-TILF had a significantly higher mean DH at post-operation (9.02 ± 0.5 mm EXP-IFC vs. 8.08 ± 1.08 mm NE-IFC, *p* = 0.024) and at 1 year follow-up (8.92 ± 0.51 mm EXP-IFC vs. 7.96 ± 0.96 mm NE-IFC, *p* < 0.01). In a typical case, x-rays reviewed at 1 week and 1 month postoperatively shown in figure 2 demonstrated effective improvement of the DH and FH compared to the preoperative period (Figure 2). After 1 year of follow-up, the results of either EXP-IFC or NE-IFC showed that the subsidence of most cage positions was not significant. In terms of FH on the operative side, the patients who used the EXP-IFC and the patients who used the NE-IFC had no statistical significance at post-operation (19.73 ± 1.63 mm EXP-IFC vs. 18.23 ± 2.07 mm NE-IFC, *p* = 0.022) and at 1 year follow-up (17.67 ± 2.29 mm EXP-IFC vs. 16.01 ± 2.73 mm NE-IFC, *p* = 0.042). Interestingly, for FH on the contralateral side, the patients who used the EXP-IFC had a significantly higher mean FH at post-operative (19.08 ± 2.40 mm EXP-IFC vs. 17.84 ± 2.21 mm NE-IFC, *p *= <0.01) and at 1 year follow-up (17.32 ± 2.26 mm EXP-IFC vs. 16.10 ± 2.32 mm NE-IFC, *p* < 0.01). No significant differences in LL were observed between patients with EXP-IFC and NE-IFC at 1 year follow-up (28.30 ± 8.43 mm EXP-IFC vs. 27.74 ± 6.08 mm NE-IFC, *p* = 0.823). Similarly, different type of the implants did not contribute to changes in SL (Table 4).

**Table 4:** Radiographic outcomes at pre-operation, post-operation and last follow-up between patients instrumented with EXP-IFC and NE-IFC.

Variable	Non-expandable	Expandable	*p* value
DH (mm)			
Pre-operation	5.61 ± 0.99	5.37 ± 0.84	0.431
Post-operation	8.08 ± 1.08	9.02 ± 0.55	**0**.024*
Last follow-up	7.96 ± 0.96	8.92 ± 0.51	**<0**.001*
FH on the operative side (mm)			
Pre-operation	13.46 ± 2.91	13.34 ± 2.51	0.481
Post-operation	18.23 ± 2.07	19.73 ± 1.63	**0**.022*
Last follow-up	16.01 ± 2.73	17.67 ± 2.29	**0**.042*
FH on the contralateral side (mm)			
Pre-operation	14.04 ± 2.55	13.89 ± 2.47	0.404
Post-operation	17.84 ± 2.21	19.08 ± 2.40	**<0**.001*
Last follow-up	16.10 ± 2.32	17.32 ± 2.26	**<0**.001*
LL (°)			
Pre-operation	28.09 ± 8.60	28.91 ± 8.61	0.771
Post-operation	27.45 ± 7.68	27.65 ± 9.15	0.944
Last follow-up	27.74 ± 6.08	28.30 ± 8.43	0.823
SL (°)			
Pre-operation	8.37 ± 2.39	8.41 ± 1.94	0.972
Post-operation	9.58 ± 2.24	9.84 ± 2.86	0.761
Last follow-up	11.33 ± 2.91	10.44 ± 2.43	0.312

DH, disc height; FH, foramen height; LL, lumbar lordosis; SL, segmental lordosis.

* Bold values are statistically significant *p* < 0.05.

### Complication

For EXP-IFC used in MIS-TLIF, postoperative complications occurred in 3 cases, of which 1 case was cerebrospinal fluid leakage, which was cured by head and foot high position and dressing pressure bandaging. The other 2 cases had transient lower limb weakness after operation and were discharged from hospital after 1 month of nerve nutrition and enhanced functional exercise. For NE-IFC used in MIS-TLIF, Postoperative complications occurred in 3 cases: temporary aggravation of nerve root symptoms, which was related to intraoperative nerve root traction, improved after symptomatic treatment such as dehydration and detumescence, and mild low back pain remained in some cases after follow-up.

## Discussion

Previous studies showed that restoration of sagittal alignment improve clinical results (19, 20). This study showed generally favorable radiographic outcomes at 1 year follow-up after EXP-IFC had been implanted in MIS-TLIF for patients with LBP or unilateral lower limb symptoms with or without contralateral lower limb mild symptoms. The use of EXP-IFC achieved 2 better radiographic objectives: significantly higher mean DH and on the contralateral higher mean FH compared with the NE-IFC in MIS-TLIF. In our study, there were no differences in OT, EBL, LOS, identified for MIS-TLIF between two groups.

Interbody fusion can rebuild the spinal stability and make the abnormal load correction, so the selection of intervertebral support materials was important in ensuring and improving the operation curative effect (21). Moreover, it has been reported that forcing a cage into intervertebral space results in collapse of the cage and the disc, the incidence of which can significantly decreased by choosing a smaller cage that is expandable after implantation (22, 23). In order to solve the deficiency of NE-IFC, various EXP-IFCs have been developed. The size of the EXP-IFC is significantly smaller than the NE-IFC, so the use of the EXP-IFC is less invasive to dura mater and nerve root than the NE-IFC. However, there are only a few studies focusing on the prognosis of EXP-IFCs. A study by Yee et al. (24) reviewed 89 patients (48 with NE-IFCs and 41 with EXP-IFCs) with lumbar degenerative conditions undergoing TLIF, and revealed that the EXP-IFC could not contribute to improvement of SL and LL compared to the NE-IFC. Another study by Hawasli el at (25) retrospectively studied 48 patients undergoing MIS TLIF with either an EXP-IFC or NE-IFC. They revealed that EXP-IFC increased SL but did no affect overall LL. In our study, there was no difference between the two groups in terms of OT, EBL, and LOS of MIS-TLIF. Furthermore, there were no significant differences in clinical outcomes at the last follow-up between the two groups. The above results suggest that the operation time of MIS-TLIF and the amount of blood loss largely depend on the skill of the operator, and the clinical outcome was more related to the duration of the procedure, the manner of the operation and the degree of decompression, but not to the type of fusion device.

When DH is reduced to about 4 mm, especially with the loss of height at the posterior edge of the intervertebral space, the foraminal area volume decreases accordingly, which may cause nerve roots compression in the foraminal area (26). The use of interbody fusion device can effectively help to restore the DH and FH (27). A retrospective study by Avani et al. (8) found that the use of either NE-IFC or EXP-IFC in MIS-TLIF can both restore DH and maintain LL angle immediately after surgery, which were not associated with selection of EXP-IFC or NE-IFC and the postoperative SL depends primarily on the preoperative SL. In this study, it was found that the DH and FH could be well restored and the postoperative SL was larger than the preoperative, and LL could be well maintained. One year later, although it was found that DH and FH were lost to a certain extent, they were still better than the preoperative and did not affect the postoperative clinical outcomes. Because of the complete release of nerve root canal decompression during the operation, the partial loss of DH will not lead to intervertebral foramen stenosis and oppress the nerve root. Through the preoperative and postoperative imaging observation, it was found that the patients over 60 years old had relative worse stenosis of intervertebral space (the sinking of fusion cage was more obvious during the last follow-up), and the reasons may be a decrease in bone mineral density in elderly patients such as osteoporosis.

The main complications of vertebral body fusion include nerve root or cauda equina injury, dural tear, intervertebral space infection, fusion cage displacement or prolapse, intervertebral bone graft non-fusion, loss of intervertebral space height and adjacent vertebral body degeneration instability and so on (28). In this study, there was no significant difference in the incidence of complications between patients with EXP-IFC and NE-IFC, which may be related to insufficient sample size and insufficient follow-up time. Further studies with larger sample sizes and more comprehensive follow-up outcomes are needed to explore complications.

There are several limitations in our study. First, election bias may exist, as the choice of EXP-IFC or NE-IFC wax`s largely determined by surgeon preference and patient pathology examination. Second, postoperative complications were evaluated by possible incomplete clinical records and possible selection or information biases were introduced. Third, the analysis of subsidence is performed by lateral x-ray measurements and will be strengthened by further radiological studies. Future studies will benefit from increased CT analysis of subsidence during and after operation. Fourth, the small sample size and limited incidence of events in our study limit the correlation between the results of the study and the entire population, and increase concerns about over-fitting of regression analysis. Finally, the mean follow-up time of 1 year is relatively short and long-term follow-up is needed to further explore the effects of EXP-IFC.

## Conclusion

In conclusion, EXP-IFC that support interbody fusion is less invasive than NE-IFC in MIS-TLIF and the use of EXP-IFC is better for the restoration of disc height and intervertebral foramen height, which caters to the current minimally invasive concept of spinal surgery. Thus, the increased cost of EXP-IFC maybe reasonable due to the pathology requirement of greater foraminal decompression. EXP-IFC has a good clinical application prospect, and is worthy of further research and application.

## Data Availability

The original contributions presented in the study are included in the article/Supplementary Materials, further inquiries can be directed to the corresponding author/s.
